# A Rare Case of Hemophagocytic Lymphohistiocytosis in an Adult with Diffuse Large B-Cell Lymphoma

**DOI:** 10.1155/2019/7530698

**Published:** 2019-07-07

**Authors:** Chimezie Mbachi, Rita Igwilo, Estefania Flores, Ezekiel Chukwujindu, Benjamin Mba

**Affiliations:** ^1^Internal Medicine, John H. Stroger Jr. Hospital of Cook County, Chicago, IL 60612, USA; ^2^Internal Medicine, University of Nigeria Teaching Hospital, Ituku-Ozalla, Enugu, Nigeria

## Abstract

A 71-year-old Indian female presented with a 3-month history of weight loss and fatigue. Further review confirmed a histological diagnosis of diffuse large B-cell lymphoma. Although bone marrow analysis did not reveal hemophagocytosis, she had some clinical and laboratory pointers to hemophagocytic lymphohistiocytosis (HLH). Her clinical state deteriorated rapidly with development of acute respiratory distress syndrome, diffuse alveolar hemorrhage, and subsequently death.

## 1. Background

This is a rare case of hemophagocytic lymphohistiocytosis in an elderly woman with diffuse large B-cell lymphoma. This case, culminating in the patient's demise, highlights the nonspecific, varied presentation and rapidly progressive nature of untreated HLH.

## 2. Case Presentation

The patient was a 71-year-old woman, originally from India, who came to the United States two weeks prior to presentation to our facility's outpatient clinic with a 3-month history of weight loss, dyspepsia, and fatigue. Initial workup showed a hemoglobin level (Hb) of 9.3 g/dl (12.9–16.8 g/dl) and atrophic gastritis on esophagogastroduodenoscopy (EGD). Hemoglobin estimation done at a follow-up visit with her primary care physician 2 weeks later was found to be 5 g/dl, and hospital admission for further evaluation and treatment was recommended. She was admitted the next day through the Emergency Department of a facility closer to her home of residence where she was found to have normal vital signs; a computed tomography (CT) scan of her abdomen and pelvis showed multiple abdominal and retroperitoneal lymph nodes but none greater than 1 cm, and it also revealed multiple hypodensities in the spleen and hepatomegaly. Hemoglobin and platelet levels had a nadir of 4.6 g/dl and 1000 cells/Ul (161,000–369,000 cells/Ul), respectively, requiring several packed red cells and platelet transfusions. Other significant investigation findings were new alanine aminotransferase (ALT) and aspartate aminotransferase (AST) elevations from 31 U/l to 144 U/l (5–35 IU/l) and 52 U/l to 106 U/l (0–40 IU/l), respectively, lactate dehydrogenase (LDH) of 472 U/l (85–210 U/l), ferritin of 2,604 ng/ml (11–206 ng/dl), haptoglobin of 22 mg/dl (43–215 mg/dl), and triglycerides of 489 mg/dl (30–150 mg/dl). Hepatitis B, C, and human immunodeficiency virus (HIV) antibodies were negative. Also, anti-nuclear antibody (ANA) and Coombs' test were negative. The bilirubin level was within the reference range. Similarly, serum electrophoresis was normal. Bone marrow biopsy done at this hospital before transfer to our facility showed B cells with no evidence of monoclonality, T cells with a normal CD4 : CD8 ratio, and no diagnostic aberrant antigenic expression; blasts were not increased.

The patient was subsequently transferred to our hospital ten days after the first admission for further management. Vital signs on admission were Bp 138/73, PR 78, RR 20, and T 98.2°F. Examination revealed multiple nontender cervical lymphadenopathy. Investigations showed Hb 5 gm/dl, white blood cell (WBC) 4,200 cells/Ul, ferritin 2331 ng/ml, platelets 19,000 cells/Ul, and triglycerides 489 mg/dl. Given the high suspicion of a lymphoproliferative disorder, pathology slides (from same bone marrow biopsy done in the previous facility) were sent to a tertiary hospital for further analysis).

## 3. Outcome and Follow-Up

Hospital course was eventful as the patient became febrile on day 2 of admission, T-max 103°F. On day 3, she was started on dexamethasone due to the high suspicion of HLH at this time. On day 4 of admission, pathology results from the tertiary hospital showed CD 20+ diffuse large B-cell lymphoma. On day 6 of admission, she became progressively dyspnoeic with respiratory rate (RR) 40 cycles/min and SaO_2_ 78% on room air with increasing oxygen requirement, requiring transfer to the MICU where she was subsequently intubated with large amounts of blood exuding from the endotracheal tube after intubation. Patient's clinical status continued to deteriorate, and after careful considerations and discussions with her family, her code status was changed to “do not resuscitate” (DNR). She was subsequently extubated after which she expired on day 7 of admission.

## 4. Discussion

Hemophagocytic lymphohistiocytosis (HLH) is a life-threatening syndrome of multiorgan clinical and laboratory abnormalities due to abnormal immune activation [1]. The primary form, more common in children, is caused by mutations that affect lymphocyte cytotoxicity and the regulation of the immune system. The secondary form, also called acquired HLH, most frequently occurs in adults [2]. Triggers of the acquired form include infections (especially Epstein–Barr virus infection), immune deficiency states, lymphomas, and other malignancies [1]. Sometimes, specific genetic mutations may be found in patients with HLH. The underlying feature is immune dysregulation which results in impaired function of cytotoxic T lymphocytes (CTL) and natural killer (NK) cells, leading to an excess of activated macrophages and, consequently, a cytokine storm. Cytokines that have been implicated include interferon gamma (IFN-gamma), tumor necrosis factor alpha (TNF-alpha), and interleukins (IL) such as IL-2, IL-6, IL-10, IL-12, and IL-16 [3–5]. These lead to destruction of both diseased and normal host cells and tissues.

The clinical features of HLH are quite diverse, but it generally presents as a febrile illness associated with multiple organ involvement. HLH can be diagnosed if 5 out of these 8 diagnostic criteria are met: fever (temperature ≥38.5°C), splenomegaly, low or absent NK activity, peripheral cytopenia (at least two of the following: Hb <9 g/dl, platelet count <100,000/Ul, and absolute neutrophil count <1000/Ul), hypertriglyceridemia (fasting triglycerides >265 mg/dl) or hypofibrinogenemia (<150 mg/dl), hyperferritinemia (>500 ng/ml), sIL2R ≥2,400 U/ml, and hemophagocytosis in the spleen, liver, bone marrow, or lymph. In adults, it is not always required that these criteria be met before initiation of treatment due to the high mortality of HLH [1]. Hemophagocytosis is neither pathognomonic of nor required for the diagnosis of HLH. This feature was absent in the bone marrow biopsy result of our patient. Following serial bone marrow evaluations, however, hemophagocytosis may eventually be seen. Other sites such as the spleen, liver, and lymph nodes should also be assessed for hemophagocytosis [1]. Prior to being transferred to our facility, our patient had 4 out of 8 criteria (bicytopenia, hyperferritinemia, hypertriglyceridemia, and hepatomegaly). On the 2^nd^ day of admission in our facility, she developed the 5^th^ (fever) diagnostic criteria and was started on dexamethasone, but soon after, she developed fatal acute respiratory distress syndrome with diffuse alveolar hemorrhage. HLH can affect any organ including the lungs, liver, kidneys, and brain. In our patient, lung parenchymal injury resulted in diffuse alveolar hemorrhage which manifested as acute respiratory distress syndrome, hemoptysis, and diffuse patchy opacities on lung imaging. Similarly, she had liver involvement with hepatomegaly and deranged liver enzymes.

The H-score is an attempt to improve diagnosis of HLH. Points are given for immunosuppression; fever; organomegaly; levels of triglycerides, ferritin, alanine aminotransferase, and fibrinogen; degree of cytopenia; and presence of hemophagocytosis on the bone marrow aspirate. An H-score ≥250 confers a 99 percent probability of HLH, whereas a score of ≤90 confers a <1 percent probability of HLH [6]. Our patient's H-Score was 221, with a 96.5% probability of HLH. Also, in adult patients with a high clinical suspicion for HLH, sIL2R levels ≥2400 U/ml have been found to have an 83% sensitivity and a 100% specificity for HLH diagnosis [7], and an elevated sIL2R/ferritin ratio is also highly predictive of lymphoma-associated HLH. Although these markers are invaluable in HLH diagnosis, they are not, per se, diagnostic confirmation for HLH and must be interpreted in the right clinical context.

This case highlights the need for a high index of suspicion for HLH. Prompt treatment is critical, but the greatest barrier to a successful outcome is a delay in diagnosis due to the rarity of this syndrome, variable clinical presentation, and lack of specificity of the clinical and laboratory findings. Aggressive courses of immunosuppressants and anti-inflammatory agents (such as glucocorticoids) are used to quell the widespread inflammation. In retrospect, the index patient may have benefited from HLH-specific therapy early on in her presentation, given the suspicion of a lymphoproliferative disorder—despite initial negative biopsy results—and evidence of organ failure. In addition, employment of the sIl2R/ferritin ratio may have aided more prompt diagnosis as it has been shown to be elevated in lymphoma-associated cases of HLH. Antimicrobials are also given for the opportunistic infections that are common in these patients. Patients with lymphoma-associated HLH, as in this case, benefit the most from etoposide-containing regimens and steroids [8–10]. Etoposide selectively depletes activated T cells [11]. On November 20, 2018, the FDA approved the anti-IFN-gamma monoclonal antibody, emapalumab (proprietary name Gamifant), for salvage treatment of primary HLH in pediatric and adult HLH patients [12]. Further guidance on HLH diagnosis and treatment in adults can be seen in the recent Histiocyte Society guideline for HLH in adults [2].

Prognosis of HLH varies. Survival is dependent upon prompt recognition and treatment. Bone marrow transplant can be curative.

## Additional Points

(i) The mortality rate of HLH without treatment is high, and HLH patients with associated malignancy suffer worse prognosis. (ii) High index of suspicion, early diagnosis, and early commencement of treatment are key to survival. (iii) Therapy is both supportive and definitive, targeted at the specific organ(s) involved and the underlying condition (for example, specific treatment for malignancy). (iv) Targeted HLH therapy with emapalumab has been approved for salvage treatment of primary HLH in both adult and pediatric patients. (v) HLH therapy, including stem cell transplant, has revolutionalized treatment and can result in long-term survival and cure.
